# Supplementary figures and images for: Asthma Care from Home: Study protocol for an effectiveness-implementation evaluation of a virtually enabled asthma care initiative in children in rural NSW
Source: PLoS One. 2024 Jun 13;19(6):e0304711. doi: 10.1371/journal.pone.0304711 (PMC11175534; doi:10.1371/journal.pone.0304711)

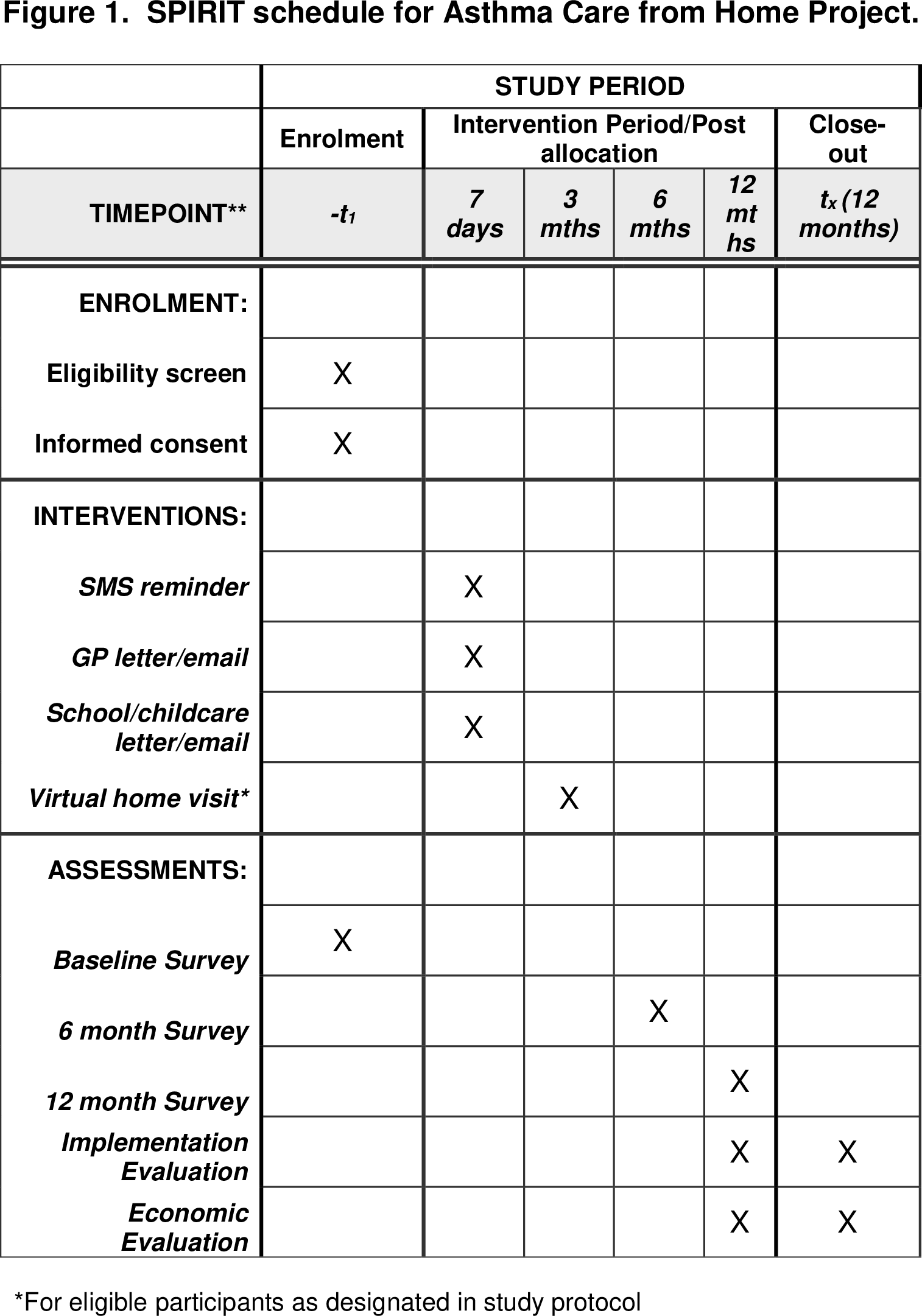

Supplement: S1 Fig — (TIF) [file pone.0304711.s001.tif]

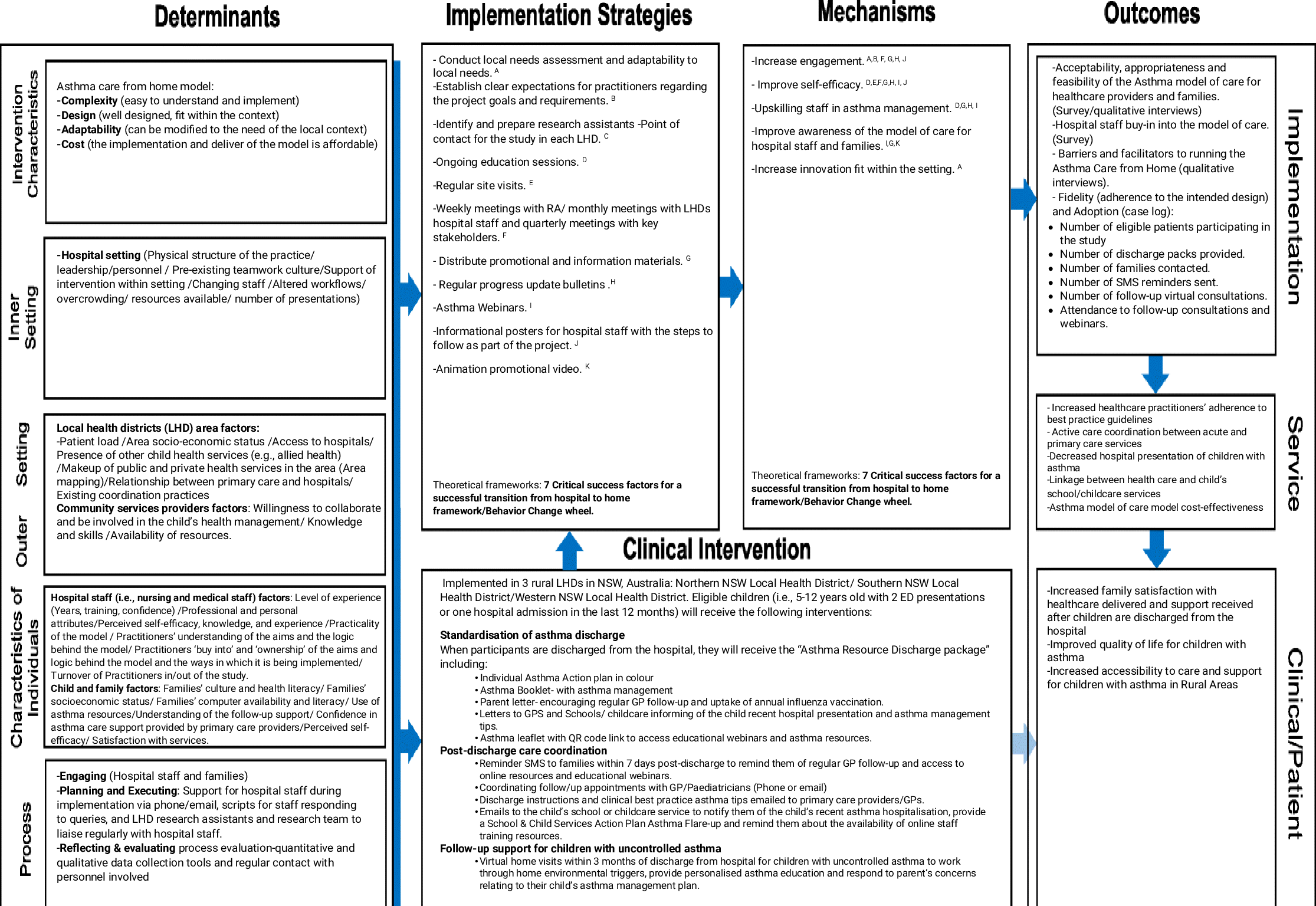

Supplement: S2 Fig — (TIF) [file pone.0304711.s002.tif]

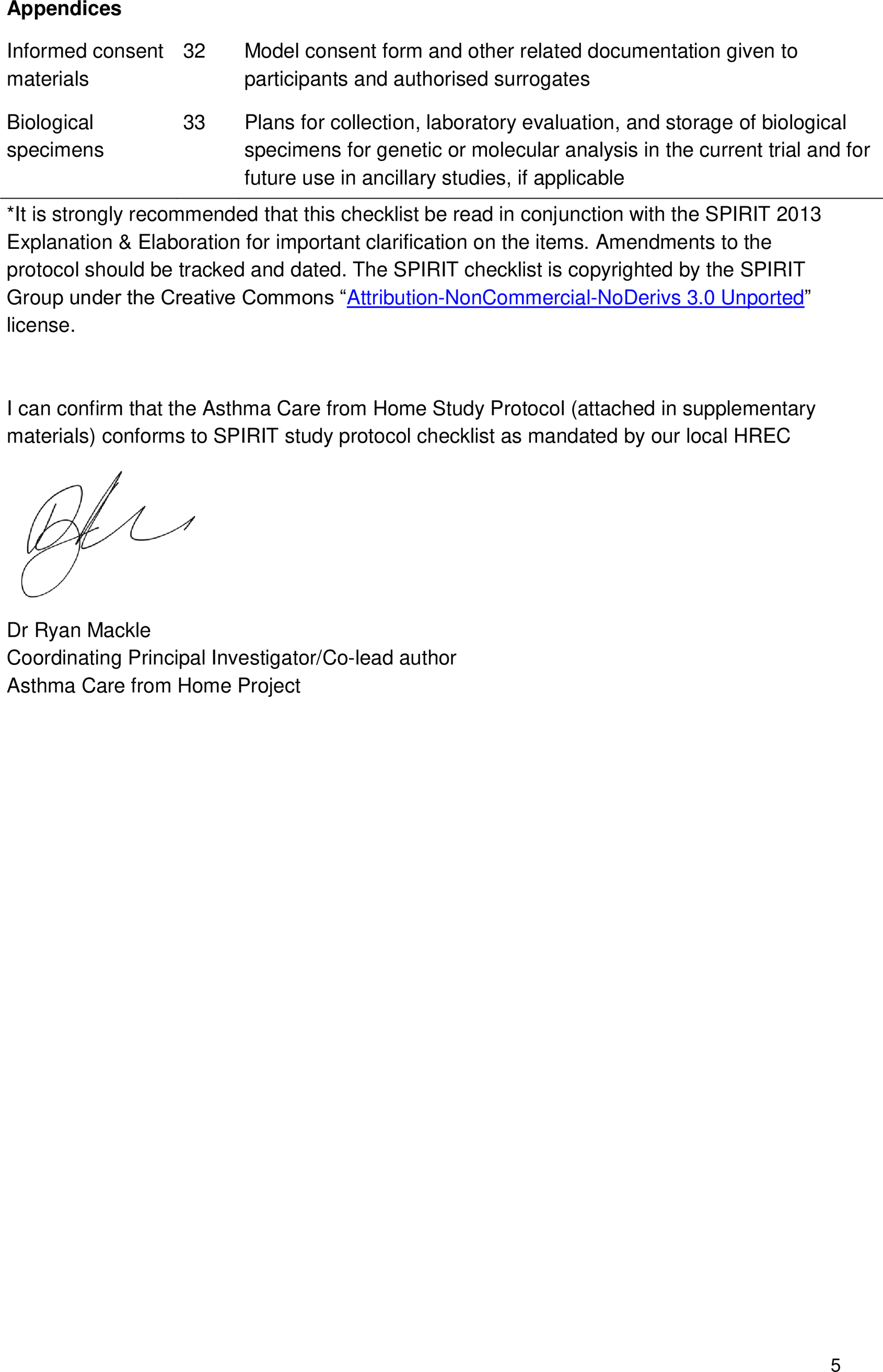

Supplement: S3 Fig — (TIF) [file pone.0304711.s003.tif]
