# Supplementary material for: Asthma Care from Home: Study protocol for an effectiveness-implementation evaluation of a virtually enabled asthma care initiative in children in rural NSW
Source: PLoS One. 2024 Jun 13;19(6):e0304711. doi: 10.1371/journal.pone.0304711 (PMC11175534; doi:10.1371/journal.pone.0304711)
Supplement: Attachment — Submitted filename: Response to Editors Dec 2023.docx [file pone.0304711.s004.docx]

**Dr Ryan Mackle and Dr Carmen Crespo-Gonzalez**
 Discipline of Paediatrics and Child Health,

UNSW Sydney
 Level 8, The Bright Alliance,

Cnr High Street and Avoca Street

Randwick NSW 2031, Australia

7^th^ December 2023

Dear PLOS One Editorial team.

**RE: Response to edits requested on PONE-D-23-36574**

We appreciate the feedback received on our manuscript submission (PONE-D-23-36574). Regarding the primary query requiring submission of a trial registry number, our Human Research Ethics Committee (Sydney Children’s Local Health District HREC) did not require prospective registration with Australia and New Zealand Clinical Trials Registry (ANZCTR) as they did not judge our project as a “clinical trial”. As such we have not registered this project with a trial registry. We are still very keen to publish our study protocol manuscript in PLOS One so would appreciate your guidance on how to proceed.

Copies of SPIRIT checklist, SPIRIT schedule, original ethics approval letter (including ethics approved study protocol document) and an updated cover letter have been uploaded and attached. Recruitment and data collection will not be completed until May 2025 so we believe this manuscript is still suitable as a study protocol article type

Thank you for your consideration

Sincerely

Dr Ryan Mackle and Dr Carmen Crespo-Gonzalez
