# Supplementary material for: Asthma Care from Home: Study protocol for an effectiveness-implementation evaluation of a virtually enabled asthma care initiative in children in rural NSW
Source: PLoS One. 2024 Jun 13;19(6):e0304711. doi: 10.1371/journal.pone.0304711 (PMC11175534; doi:10.1371/journal.pone.0304711)
Supplement: Attachment — Submitted filename: Response to Reviewers April 2024.docx [file pone.0304711.s005.docx]

26^th^ April 2024

**Dear Editor,**

**We are grateful to the reviewers for their review of our original protocol paper titled “Asthma Care from Home: Study protocol for an effectiveness-implementation evaluation of a virtually enabled asthma care initiative in children in rural NSW”. We have addressed the comments raised by reviewers and below are our point-by-point responses.**

**ACADEMIC EDITOR RESPONSE**

**1. Please ensure that your manuscript meets PLOS ONE's style requirements, including those for file naming.**

The revised manuscript has been amended to ensure it fulfils PLOS ONE’s style requirements.

**2. We note that you have selected “Clinical Trial” as your article type. PLOS ONE requires that all clinical trials are registered in an appropriate registry (the WHO list of approved registries is at      https://www.who.int/clinical-trials-registry-platform/network/primary-registries"" https://www.who.int/clinical-trials-registry-platform/network/primary-registries and more information on trial registration is at http://www.icmje.org/about-icmje/faqs/clinical-trials-registration/).
 
Please state the name of the registry and the registration number (e.g. ISRCTN or ClinicalTrials.gov) in the submission data and on the title page of your manuscript.**

**a) Please provide the complete date range for participant recruitment and follow-up in the methods section of your manuscript.**

**Authors response:**

Thank you for your comment. The methods section has been updated according to the Editor’s suggestion.

**Change(s) in manuscript:**

*Methods section, Line 172-175:*

“Between March 2023 and June 2024, children aged between 5-12 years old presenting to ED on one or more occasions or admitted to the general paediatric ward on one or more occasion in the last 12 months with asthma at selected study hospital sites within the three LHDs will be eligible to participate in the study.”

**b) If you have not yet registered your trial in an appropriate registry, we now require you to do so and will need confirmation of the trial registry number before we can pass your paper to the next stage of review. Please include in the Methods section of your paper your reasons for not registering this study before enrolment of participants started. Please confirm that all related trials are registered by stating: “The authors confirm that all ongoing and related trials for this drug/intervention are registered”.
 
Please see http://journals.plos.org/plosone/s/submission-guidelines#loc-clinical-trials for our policies on clinical trials**

**Authors response:**

Previous correspondence detailed that clinical trial registration was not required for publication of our study protocol paper. Email thread containing this documentation has been submitted as supporting evidence (Supporting Evidence - PLOS ONE PONE-D-23-36574 - EMID6d5e96dff22f4957).

**3. We note that the grant information you provided in the ‘Funding Information’ and ‘Financial Disclosure’ sections do not match.**

**When you resubmit, please ensure that you provide the correct grant numbers for the awards you received for your study in the ‘Funding Information’ section.**

**Authors response:**

We apologise for the confusion around Funding and Financial Disclosures. This study was funded by a grant from the NSW Health Translational Research Grants Scheme. These are not provided with a designated “grant number”. Our funding disclosure statement has been included in both Funding and Financial Disclosures section. RM is currently undertaking a PhD that is supported by scholarships from the Australian Government Research Training Program (RTP), Asthma Australia and the Zoe Kennedy Foundation. The PhD scholarship funding has therefore only been included within the financial disclosures section of the manuscript.

**4. Thank you for stating the following financial disclosure: 
"This project was funded by a grant from the NSW Health Translational Research Grants Scheme. The views expressed are those of the authors and not necessarily those of the partner organisation. RM is supported by PhD scholarships from the Australian government Research Training Program (RTP), Asthma Australia and the Zoe Kennedy Foundation. "**

**Please state what role the funders took in the study.  If the funders had no role, please state: ""The funders had no role in study design, data collection and analysis, decision to publish, or preparation of the manuscript."" 
If this statement is not correct you must amend it as needed. 
Please include this amended Role of Funder statement in your cover letter; we will change the online submission form on your behalf.**

**Authors response:**

We have amended the financial disclosure statement with this submission. Please see cover letter.

**5. Please include your full ethics statement in the ‘Methods’ section of your manuscript file. In your statement, please include the full name of the IRB or ethics committee who approved or waived your study, as well as whether or not you obtained informed written or verbal consent. If consent was waived for your study, please include this information in your statement as well.**

**Authors response:**

Thank you for your comment. The full ethics statement has now been added to the ‘Methods’ section of the revised manuscript according to the Editor's suggestions.

**Change(s) in manuscript:**

*Study design and setting section, Line 166-170:*

“Ethical approval was granted by the Sydney Children’s Hospital Network Human Research Ethics Committee in November 2022 (2022/ETH01946). Site specific Research Governance was granted across all participating Local Health District Research Governance by March 2023 (2022/STE03422, 2022/STE03423, 2022/STE03424 and 2022/STE03425).”

*Study population section, Line 184-186:*

“Informed written consent was sought from parents/carers of eligible children electronically via REDcap software.”

**6. We note that Figure 1 in your submission contain map image which may be copyrighted. All PLOS content is published under the Creative Commons Attribution License (CC BY 4.0), which means that the manuscript, images, and Supporting Information files will be freely available online, and any third party is permitted to access, download, copy, distribute, and use these materials in any way, even commercially, with proper attribution. For these reasons, we cannot publish previously copyrighted maps or satellite images created using proprietary data, such as Google software (Google Maps, Street View, and Earth). For more information, see our copyright guidelines: http://journals.plos.org/plosone/s/licenses-and-copyright.
 
We require you to either (1) present written permission from the copyright holder to publish these figures specifically under the CC BY 4.0 license, or (2) remove the figures from your submission:
 
a. You may seek permission from the original copyright holder of Figure 1 to publish the content specifically under the CC BY 4.0 license.  
  
b. If you are unable to obtain permission from the original copyright holder to publish these figures under the CC BY 4.0 license or if the copyright holder’s requirements are incompatible with the CC BY 4.0 license, please either i) remove the figure or ii) supply a replacement figure that complies with the CC BY 4.0 license. Please check copyright information on all replacement figures and update the figure caption with source information. If applicable, please specify in the figure caption text when a figure is similar but not identical to the original image and is therefore for illustrative purposes only.**

**Authors response:**

The map included in our original manuscript was adapted from a map included in a previous journal manuscript submitted by our team (Chan et al, Journal of Asthma and Allergy 2021 (DOI: 10.2147/jaa.s311721)). As it does not fulfil PLOS One guidelines, we have removed it from our revised manuscript.

**7. Please include captions for your Supporting Information files at the end of your manuscript, and update any in-text citations to match accordingly. Please see our Supporting Information guidelines for more information: http://journals.plos.org/plosone/s/supporting-information.**

We have made the requested amendments to Supporting Information

**8. Please review your reference list to ensure that it is complete and correct. If you have cited papers that have been retracted, please include the rationale for doing so in the manuscript text, or remove these references and replace them with relevant current references. Any changes to the reference list should be mentioned in the rebuttal letter that accompanies your revised manuscript. If you need to cite a retracted article, indicate the article’s retracted status in the References list and also include a citation and full reference for the retraction notice.**

**Authors response:**

Thank you for your comment. The reference lists have been reviewed and updated to improve accuracy.

**Change(s) in manuscript:**

*Reference list section:*

1 Global Asthma Network. The Global Asthma Report 2022 Auckland. New Zealand: Global Asthma Network; 2022. [cited 2022 28 August]. Available from: [www.globalasthmareport.org/resources/global_asthma_report_2022.pdf](http://www.globalasthmareport.org/resources/global_asthma_report_2022.pdf).

*Changed to*

1. Global Asthma Network. The Global Asthma Report 2022. Auckland, New Zealand 2022 [cited 2024 26th April]. Available from: <http://globalasthmareport.org>

2 Global Asthma Network. The Global Asthma Report 2018 Auckland. New Zealand: Global Asthma Network; 2018. [cited 2023 28 August]. Available from: [www.globalasthmareport.org/resources/global_asthma_report_2018.pdf](http://www.globalasthmareport.org/resources/global_asthma_report_2018.pdf)

*Changed to*

2. Global Asthma Network. The Global Asthma Report 2018. Auckland, New Zealand 2018 [cited 2024 26th April]. Available from: <http://globalasthmareport.org/2018/index.html>

6. HealthStats. NSW Population by remoteness, age and year 2020

*Changed to*

Centre for Epidemiology and Evidence. Asthma prevalence in children by Asthma Type and Remoteness category. Sydney: HealthStats NSW; 2020 [cited 2024 26th April]. Available from: <https://www.healthstats.nsw.gov.au/r/112635>

9. Australian Bureau of Statistics. QuickStats,. Canberra,, 2021.

Changed to:

7. Australian Bureau of Statistics. Quickstats, Australia. Canberra: Australian Bureau of Statistics; 2021.

17. NSW Health. Schools and child services action plan for asthma flare-up.: NSW Government; 2023 [Available from: <https://www.schn.health.nsw.gov.au/files/factsheets/asthma_-_schools_and_child_services_action_plan_for_asthma_flare_up-en.pdf>].

Changed to

19. NSW Health. Schools and Child Services Action Plan for Asthma Flare-up. [cited 2024 26th April]. Available from: <https://www.schn.health.nsw.gov.au/sites/default/files/2024-01/21138465_schn_asthma_flare_up_document_v3_1.pdf>

*New reference in response to reviewers comments:*

18. Department of Health and Aged Care. Influenza (Flu), Australian Immunisation Handbook. [cited 2024 26th April]. Available from: <https://immunisationhandbook.health.gov.au/>

20. Casale TB, Pedersen S, Rodriguez Del Rio P, Liu AH, Demoly P, Price D. The Role of Aeroallergen Sensitization Testing in Asthma Management. J Allergy Clin Immunol Pract 2020;8(8):2526-32

35.Weiner BJ, Lewis CC, Stanick C, Powell BJ, Dorsey CN, Clary AS, Boynton MH, & Halko H. Psychometric assessment of three newly developed implementation outcome measures. Implementation Science, 2017;12(108), 1-12.

**REVIEWER RESPONSE**

**Reviewer #1:**

**Study is New in its concept for the individual family and the community. But without results of data analysis how can reviewer knows the effect/impact of such community-based study. Control group may be considered to observe the effect whether increasing decreasing or remaining same from the same community without home-based management.**

**Please mention the limitation of the study**

**Mention the exclusion criteria**

**how the author has avoided the selection bias.**

**Authors response:**

We appreciate the reviewer’s comment. As this is a study protocol paper, hence does not describe any result, this paper outlines how the study will be conducted. We will have the results once the study is completed. We do accept that the one-group pre-post-trial design limits our ability to assess and compare the effects of our initiative with usual care, however in consultation with our LHD partners, a randomised controlled trial was deemed unsuitable due to the standardised nature of the intervention. Instead, we chose a pre/post quasi-experimental study design whereby each participant acts as their own control which minimises selection bias. As we are including all participants who meet eligibility criteria, convenience and random sampling are not indicated. Exclusion criteria is available in the Methods section [Line 196-203 original manuscript] and study limitations available in the Discussion [Line 517-526 original manuscript].

**Reviewer #2:**

**The authors of the manuscript present here the design of a quasi-experimental pre- and post-interventional trial for an asthma care from home project. This ambitious study specifically aims to improve access to asthma care for affected children and their families in rural and remote areas in Australia.**

**The manuscript is written with excellent expression and spelling, making it easily understandable. The authors based their design on substantial experience in former studies. Each part of the design is explained in detail and decisions made for or against certain proceedings are discussed and made transparent. One minor point from my side is to ask the authors to shortly explain why encouragement for uptake of annual influenza vaccination is implemented in the parent letter. Despite of the that, I have no further remarks.**

**Authors response:**

We thank Reviewer 2 for their valuable feedback. Annual influenza vaccination is recommended in international guidelines due to the increased risk of morbidity and mortality associated with influenza infection in asthmatics. The Australian Immunisation Handbook also recommends seasonal influenza vaccination for children with asthma. The manuscript has been amended to make our rationale clearer.

**Change(s) in manuscript:**

*Study intervention section, Line 209-211:*

“…a parent letter encouraging regular GP follow-up and uptake of annual influenza vaccination (as recommended in GINA guidelines [17] and Australian Immunisation Handbook [18].”

**Reviewer #3:**

**I. The authors provide a detailed outline of a study designed to establish a state wide program of at home asthma management for families living in rural areas. The current manuscript has two obvious problems:**

**i) it is too long and, in many places, repetitive.**

**ii) In many places the language is verbose. A particular example comes in the second page of the discussion (page 2). To this reviewer, the sentence starting on line 14 with “Moreover it facilitates” includes a remarkable number of excessive words! [Line 514-517]**

**Authors response:**

We appreciate Reviewer 3’s feedback. We have updated the manuscript to make it more concise.

**Change(s) in manuscript:**

*Study interventions section, Line 205-211:*

“Participants discharged from the hospital will receive the “Asthma Resource Discharge package” which contains several pre-developed resources including an individualised asthma action plan (AAP) in colour [16], an asthma education booklet entitled “Asthma and Your Child – a resource pack for parents and carers” to improve parent’s/carer’s understanding of asthma that is available in multiple languages, a parent letter encouraging regular GP follow-up and uptake of annual influenza vaccination (as recommended in Global Initiative for Asthma (GINA) guidelines [17] and the Australian Immunization Handbook [18])”

*Study interventions section, Line 222-231:*

“Parents/carers of the participating child will firstly receive a text message (SMS) reminder within 7 days of hospital discharge to remind them of regular GP follow-up and provide links to online resources and links to register for monthly educational webinars with the AAIC. In addition, local investigators will coordinate follow-up appointments with GPs and/or paediatricians through telephone or email to facilitate tele-consultation where possible and provide discharge instructions. Similarly, the child’s school or childcare service will be notified by email of the child’s recent asthma hospitalisation, provided an updated School & Child Services Action Plan ((NSW Health)) and reminded of the availability of online staff training resources including the AAIC eBook on Asthma First Aid in Children or training sessions for childcare staff via ACECQA (Australian Children’s Education & Care Quality Authority).”

*Implementation evaluation- Surveys section, Line 380-381:*

“The survey will include a validated tool, based on the theoretical framework of acceptability (TFA) of health interventions tool [32]”.

*Line 385-388:*

" The survey will include two validated instruments: the short intervention acceptability, appropriateness and feasibility measure, and the NOMAD tool (Table 3) [33,34,35]. Items will be rated on a 5-point Likert scale from 1 (strongly agree) to 5 (strongly disagree).”

*Discussion section, Line 481-486:*

“Our model of care offers a solution to the current fragmented and inadequate Australian healthcare system. This model aims to standardise the care provided for children with asthma and provide easy access to in-depth educational resources and support tools for families, healthcare providers and community services so a child’s asthma can be managed effectively outside of a hospital setting and closer to home.”

*Line 487-492:*

“We have used a pre-post trial design, which will allow us to determine the changes in health services used due to our model of care. The pre-intervention period of health service utilisation for each participant will be incorporated as a temporal control. A notable strength of this design is that since the same group serves as its control, it can help control for individual differences that might confound the results in studies involving separate control groups”

*Line 502-508:*

“An added advantage of our methodology is integrating a theory-informed mixed-method process evaluation. This inclusion allows us to comprehensively grasp the obstacles and enablers of implementing this initiative within real-world practice, and facilitates the identification of strategies conducive to its sustained implementation [40]. Concurrently, we will undertake an economic evaluation to establish a viable rationale for healthcare organisations to allocate resources towards adopting the Asthma model of care. This will substantiate the tangible advantages of such an uptake on the Australian healthcare system.”

*Line 519-525:*

“In conclusion, this study aims to significantly enhance access to asthma care, particularly targeting children in underserved communities like those living in rural and remote areas. By assessing implementation and economic factors, our research will provide comprehensive understanding of the effectiveness and viability of the model to help integrate it into standard practice. By utilising local resources, we can ensure the sustainability of improved healthcare services and help shed light on the potential for scaling up this model to benefit a wider population of children facing asthma-related challenges in underserved regions.”

**II. The map does not explain the two colors if it is just to distinguish different regions it needs to say so. To a non-Australian the map would be more useful if the towns were indicated, i.e., Sydney, Newcastle, Waga-Waga, Townsville, and Brisbane.**

**Authors response:**

Thank you for your comment. We have removed the map based on the editors’ recommendation.

**III. There is a mention of environmental assessment but no references to the major studies on the relevance of environment, e.g., dust mites: including studies by Ann Woolcock, Jenny Peat, Dr. Marks, and Euan Tovey. All of these worked in N.S.W.**

**Authors response:**

We appreciate the feedback. We have included a brief rationale in the methods section to explain why environmental assessment is important in children with asthma including a more recent reference from Casale et al.

**Change(s) in manuscript:**

*Study interventions section, Line 238-240:*

" Assessment of environmental triggers including aeroallergens like house dust mite, pollens and grasses is important as they are key contributors to asthma disease and can result in frequent flare-ups [20].”

**IV. Why are truly poor patients excluded? Is there a danger that those individuals will recognize that they are being excluded? How many children will be living in a shelter? What is the justification for restricting age to 5-12 years?**

**Authors response:**

We disagree that the project is excluding poor patients/children. Data on children living in sheltered accommodation, temporary residence or classified as homeless is not available. However, The Australian Institute for Health and Welfare reports at a general population level that rates of those seeking specialist homelessness services are lower in our study areas (Outer regional NSW (7%) and rural/remote NSW (1%)) [AIHW 2023, <https://www.aihw.gov.au/getmedia/1dcc1074-ce82-4732-b81e-f68b47d1fafc/hou333_factsheet_nsw.pdf> ]. As sheltered accommodation is usually temporary, we are unable to assess home environmental influences on the child’s asthma reliably. Families may also not remain within their geographical areas depending on their reason for homelessness and/or temporary accommodation, which can impact the environmental influences placed upon their child’s health and therefore the validity of our results.

Parents/carers are blinded to eligibility criteria. All individuals discharged from study hospital sites are provided with our asthma discharge resource packs, regardless of inclusion in the study which minimises discrimination against population groups.

Regarding age restriction, hospitalisation rates of children with asthma are highest in 5-9 years and 10-14 years age groups [AIHW 2022, <https://www.aihw.gov.au/reports/children-youth/australias-children/contents/health/asthma-prevalence-children> and <https://www.aihw.gov.au/reports/chronic-respiratory-conditions/asthma>]. Additionally, asthma management, and the respective involvement of the child and their parent/carer, varies by age group. The National Asthma Council Australia separates children into i) 5 years and under, ii) 6-12 years old, and iii) 12 years and over. So, confining our recruitment to 5-12 years old was deemed a logical choice.

**Minor comments**

**V. Do all subjects have to have cell phones?**

**Authors response:**

Mobile phones are not a compulsory element of this project however it would not be expected to be an issue. (Australian Mobile Telecommunications Association (AMTA) <https://amta.org.au/about-amta/#:~:text=With%209%20out%2010%20Australians,mobile%20telecommunications%20industry%20in%20Australia>; Infrastructure Australia. Mobile telecommunications coverage in regional and remote areas. Commonwealth of Australia. 2020 [www.infrastructureaustralia.gov.au/map/mobile-telecommunications-coverage-regional-and-remote-areas](http://www.infrastructureaustralia.gov.au/map/mobile-telecommunications-coverage-regional-and-remote-areas).).

**VI. The abbreviation SMS appears in the abstract without introduction.**

**Authors response:**

We have amended the abstract to describe “text message” rather than SMS.

**Change(s) in manuscript:**

*Abstract section, line 56:*

“..Post-discharge care coordination through text message reminders for families for regular GP review”.

**Reviewer #4:**

**The study protocol aims to evaluate a digitally enabled asthma care initiative for children in rural NSW. The initiative builds on prior successful models, focusing on standardizing asthma discharge, coordinating care between acute and primary services, and providing home-based follow-up. The paper appears to be well-written. However, there are a few areas where slight improvements could be made for clarity or conciseness:**

**1. Rephrase this sentence "The initiative builds on prior successful models, focusing on standardizing asthma discharge, coordinating care between acute and primary services, and providing home-based follow-up,"**

**2. The phrase "particularly in areas with limited access to care" could be expanded for clarity.**

**Authors response:**

We thank the reviewer for their comments. On review of the original submitted manuscript, we cannot identify the sentence “The initiative builds on prior successful models, focusing on standardizing asthma discharge, coordinating care between acute and primary services, and providing home-based follow-up," or the phrase “particularly in areas with limited access to care" requested for editing.

**3. The hypothesis is not correctly aligned with the study objectives and the title.**

**Authors response:**

We have amended the hypothesis to include “virtually enabled asthma model of care” to better reflect the manuscript title and study objectives.

**Change(s) in manuscript:**

*Hypotheses and Aims section, Line 141:*

“We hypothesise that a comprehensive virtually enabled asthma model of care that leverages digital technologies can provide continuity of care, improve asthma quality of life and lead to reduction in unscheduled hospital presentations in children with asthma aged 5-12 years living in rural NSW.”

**4. Sampling technique is not mentioned and how will you do the sampling.**

**Authors response:**

We will approach all parents/carers of all children meeting our inclusion and exclusion criteria. All consenting parents/carers and their children will be included in the study.

**5.** **Can it will possible to do the panel data modelling to include the effect of the study area?**

**Authors response:**

Yes, we will conduct a multi-level regression analysis with a fixed effect of intervention and a random effect of study site (i.e., the Local Health District) to explore the degree of variation of the intervention effect by site.

**Change(s) in manuscript:**

*Aim 1 Impact Evaluation, Data Analysis section, Line 332-339:*

“Since each participant will have three repeated measures over time, we will use multi-level regression analysis to examine the effect of the intervention on these outcomes, including in the models, a fixed effect for the intervention status (0=pre-intervention, 1=6-month post-intervention, 2=12-month post-intervention) and calendar time of enrolment into the intervention for each participant, as well as a random effect of the Local Health District (LHD) to explore the degree of variation in the intervention effect by LHD. Our final model will exclude the random term in the presence of a statistically non-significant random effect.”

**Reviewer #5:**

**The objective of this paper is to describe a planned intervention trial for children with asthma to improve access to asthma care for children in remote and rural areas. I commend the authors for this important work, as improving assess to care is a problem worldwide. I have a few comments on the methodology of the planned intervention:**

**1. Virtual home visits: only children with uncontrolled asthma, defined by ACT <19 will be offered the virtual home visit intervention. When will the ACT be administered to determine controlled/uncontrolled status? By definition, all kids presenting to the ED have current uncontrolled asthma and would likely have an ACT score at baseline <19. While this will likely lead to most children receiving the virtual home visit intervention, why not include all children?**

**Authors response:**

We thank Reviewer 5 for their feedback. C-ACT/ACT will be measured at enrolment and those children with C-ACT/ACT scores <19 will be offered a virtual home visit within 3 months of discharge from hospital [Line 241-242 original manuscript]. We do not agree that all kids presenting to ED have current uncontrolled asthma, as the definition of “uncontrolled asthma” is more nuanced than ED presentation alone. Recent literature has shown an increasing trend in parents/carers using Emergency Departments for primary care issues (Neill et al, Archives of Disease in Childhood 2018; Unwin et al, International Emergency Nursing 2016). Similarly, the most common trigger for asthma exacerbations in children is respiratory infections (Navanandan et al, JACI: In Practice 2021), which in school-aged children can occur on average 8 times per annum (Thompson et al, BMJ 2013; Chang et al, Chest 2012). Therefore, accounting for solely hospitalisation is unlikely to give a true representation of asthma control. For the purposes of our study, we instead classify uncontrolled asthma as children with a C-ACT/ACT <19.

Offering a virtual home visit to all participants is a valid suggestion however as asthma is the most common chronic condition in Australian children, providing a virtual home visit to every child that presents to hospital would be resource intensive and might not be a sustainable and scalable intervention in real world scenario. Even though we will not be providing virtual home visits to all children, all parents/carers are provided links to free virtual asthma education webinars which include topics about home environmental triggers and asthma medication use/techniques

**2. Please define severe asthma in the methods.**

**Authors response:**

We have amended the methods section to further clarify our definition of severe asthma. For the purposes of our study, we have used the Global Initiative in Asthma (GINA) definition of severe asthma.

**Change(s) in manuscript:**

*Methods section, Line 247-252:*

“For children identified as having severe asthma, defined by GINA as “asthma that is uncontrolled despite adherence with maximal optimized therapy and treatment of contributory factors, or that worsens when high dose treatment is decreased” (17), will also be offered joint telehealth consultation with a tertiary hospital-based paediatric respiratory consultant at the Sydney Children’s Hospital, Randwick and a locally based general paediatrician to develop a shared care plan.”

**3. Only those defined as having severe asthma will receive a telehealth consult to develop a shared asthma care plan. Why not include all children?**

**Authors response:**

We have tailored the joint telehealth consultation to fit with National Asthma Council guidelines. The Australian model of care varies from other countries e.g. the USA, in that GP specialists and General Paediatricians will manage most children with asthma (Homaira et al, Respirology 2019). Respiratory specialist review is instead for those with the most severe and uncontrolled disease, typically step 3/4 or higher on National Asthma Council management algorithm. We have amended the manuscript to make this rationale clear.

**Change(s) in manuscript:**

*Study interventions section, Line 252-254:*

“This is in keeping with National Asthma Council guidelines, which recommend that those on step 4 of asthma management algorithm be considered for specialist review (21).”

**4. Since the children receive the virtual home visit and the telehealth based on their clinical asthma status, the study will be include multiple case groups (ie controlled non-severe, controlled severe, uncontrolled non-severe, uncontrolled severe). Since not all children are receiving the same intervention package, how will the efficacy of the different packages be determined?**

**Authors response:**

Those receiving a virtual home visit are postulated to be a small group, and those receiving telehealth joint consultation are even smaller. All children will receive the remaining suite of interventions including standardised asthma discharge resource pack, 7 day SMS reminder, GP/School/Childcare letters and emails, and access to online multimedia resources. Clustering of subgroups will be accounted for in regression analysis.

**Change(s) in manuscript:**

Aim 1 Impact Evaluation, Data analysis section, Line 327-341:

“Interrupted time series (ITS) analysis will be used to assess the change in the time series patterns (intercept and slope) of health service use before and after intervention.

ACT score, asthma related quality of life, medication adherence, parent and child quality of life and family reported experiences will be compared at enrolment, 6 and 12 months after enrolment into the service. We will describe the outcomes at each time point using mean, standard deviation, median, and inter-quartile range. Since each participant will have three repeated measures over time, we will use multi-level regression analysis to examine the effect of the intervention on these outcomes, including in the models, a fixed effect for the intervention status (0=pre-intervention, 1=6-month post-intervention, 2=12-month post-intervention) and calendar time of enrolment into the intervention for each participant, as well as a random effect of the Local Health District (LHD) to explore the degree of variation in the intervention effect by LHD. Our final model will exclude the random term in the presence of a statistically non-significant random effect. We will also consider participants are clustered within each LHD and use generalised estimating equations (GEE) approach in the regression analysis to account for the clustering.”

**5. The children will be their own control, and their previous 5 years of utilization will be used to determine if the intervention leads to decreased utilization for asthma. Since the inclusionary age starts at 5 and diagnosing asthma in children under 5 is problematic, how will it be determined if utilization below age 5 is due to true asthma and not transient wheeze?**

**Authors response:**

This is a valid point. Not all enrolled children will have had healthcare utilisation in the previous 5 years. We are enrolling all children presenting to hospital with asthma, including first presenters. The impact of our interventions depending on prior healthcare utilisation rates will be accounted for in data analysis. We would propose that whether a child had transient wheeze or asthma in the last 5 years is less important, as in fact we are assessing the effect standardisation of asthma discharge and linkage with community services has upon healthcare utilisation with asthma and asthma-like conditions prospectively post intervention.

Sincerely

Dr Ryan Mackle and Dr Carmen Crespo-Gonzalez
